# Bromide Potassium in Uræmic Convulsions

**Published:** 1871-12

**Authors:** Edwin A. Carpenter

**Affiliations:** Baileyville, Ill.


### BROMIDE POTASSIUM IN URÆMIC CONVULSIONS.

By EDWIN A. CARPENTER, M. D., Baileyville, Ill.

DEAR EXAMINER: I have recently had a case in practice that may interest some of the multitude of your readers, more particularly, perhaps, the junior members thereof. I report it briefly as possible, and will be as perspicuous as I can in view of its brevity.

Oct. 29th, 1871, I was called at six A. M. to see Warren A——, æt. 17, of bilious temperament, good habits—American. His occupation is that of a farm laborer, but, for a few weeks prior to his seizure he had returned to his parents (who are residents of this village) not feeling, as he alleged, fit for the laborious duties that necessarily devolve upon those who are engaged in agricultural pursuits.

The messenger stated that he was then in a fit, and desired my immediate attendance as they feared dissolution would take place unless promptly relieved. On entering the room of the patient the first glance was to me sufficient almost for a "Diagnosis." The face was as full seemingly as the skin would hold; also, the extremities. The sides were bulged out to their fullest extent, and effusion had taken place to such an extent that it was with some difficulty that the intercostal spaces were found. Respiration was difficult and labored, so much so that the patient was obliged to keep the upright posture. Percussion elicited a dull sound to very near the apex of the lungs. Auscultation revealed heart sounds very feeble but regular, conveying a peculiar fillap, so to speak, to the ear, though by repeated subsequent examinations I am convinced that there is not any organic disease of the heart. The respiratory murmur was lost except near the apex of the lungs, and their mucous rales greeted the ear. The pulse was full but slow; tongue covered with a cream-colored fur. Cephalalgia was wanting, as also lumbar pain. Bowels constipated, and urine diminished in quantity, and had been in that condition for some time.

I diagnosed "Acute Bright's Disease," and immediately gave a powder composed as follows: Elaterium gr. 1-8; podophyllyn grs. ii. Also gave a hot air bath but was interrupted by the occurrence of another convulsion, for which I gave chloroform by inhalation, abbreviating the *duration* of the paroxysm, and have faith to believe lessened the tendency to that comatose condition which so frequently occurs in uræmic poisoning, though two distinct phenomena present themselves in this disease where convulsions take place, viz: consciousness remains if the poison acts upon the spinal cord the convulsions being of an epileptic character, frequently affecting the whole muscular system. In another form stupor supervenes; fixed pupils; stertorous breathing; in fact the symptoms are very like narcotic poisoning. Still another form belongs in this catagory. Convulsions, epileptic in character with coma.

In this case the patient was unconscious during the paroxysm, but soon regained his mental powers. In the course of half an hour he had another convulsion lasting about ten minutes in spite of a liberal use of the chloroform. I now repeated the administration of the Elaterium et Podophyllyn, also gave enemas of soap suds, believing that unless an abundant evacuation took place from the bowels soon, the patient must die, as, after repeated attempts the patient could not be made to perspire with the aid of hot air, which I consider the most potent of all cutaneous eliminants.

From the time of my arrival until 11 A. M. he had seven (7) uræmic convulsions, notwithstanding he inhaled eight ounces (fluid) of chloroform. At 10 A.M. I gave ℥i of magnesia sulphas dissolved in a glass of water, believing that I had now gone to the limits of hydragogues, providing one is to be guided by prudence. At eleven he was seized with another convulsion, after the cessation of which, I gave 40 grains of Bromide Potassium dissolved in plain water. Its soothing effects soon manifested itself though it did not cause him to sleep.

At 4 P. M. he voided an immense quantity of urine, smoky in appearance, which, upon testing by heat and nitric acid gave about four-fifths of the quantity in albumen. At 6 P. M. he had a free watery evacuation from the bowels, also passed considerable urine at the same time. At 7 P. M. I gave Brom., K ʒss. dissolved in water, after the administration of which the patient slept proped up by pillows. as it was dangerous to life to seek the recumbent posture, owing to the hydrothorax in conjunction with œdema of the lungs.

Patient passed the night comfortable comparatively speaking, having three copious evacuations from the bowels, also passed a considerable quantity of urine. On the 30th, 6 A. M., patient expressed himself as feeling first-rate, not suffering pain to the slighest extent. Pres. Iodide Potassium, grs. xv., Bromide Potassium, grs. xv., Powd. Digitalis grs. ii. Mix. To be taken at once as a diuretic and sedative to the circulation as the pulse had become frequent. Gave a hot-air bath which now induced copious perspiration. Also, pres. Magnesia sulphas ℥i., aqua pur. O., Meisce. sig. To be taken at noon. Directed as a diet: sweet skimmed milk, in quantities to suit the patient.

Six P. M. Patient had perspired all day, and to such an extent as to wet the bed to some depth. Bowels had moved several times. Kidneys responding freely. Appetite good. Pulse 76 beats per minute. Tongue not furred so much. Respiration freer. Upper part of thorax resonant on percussion. Slight cough. Again prescribed Iodide and Bromide of Potassium and Digitalis.

It will suffice to say that under eliminative treatment with tonics the patient has so far recovered that in pleasant weather he goes out of doors; his urine still showing a trace of albumen in it.

It is *not* the recovery particularly that I wish to dilate upon, but the cessation of the convulsions *after* the internal administration of the Bromide Potassium. Some may think the chloroform the efficient agent; others that the action of the powerful hydragoge-cathartics causing a transudation of fluid into the intestinal canal, in which was held the poisonous substance (urea) thereby lessening its quantity in the circulating current, was the cause of the toxic phenomena ceasing. My theory is this: It is well known that Bromide of Potassium lessens the amount of blood circulating within the cranium and spinal cord, and, as it requires a *certain amount* of urea, or if you prefer it, Carbonate of Ammonia circulating within the spinal system to produce uræmia, by lessening the amount of blood in the brain and spinal cord, we can hold the convulsions in abeyance, until we eliminate the morbid matter.

Nov. 14th. Patient has just called, stating that he felt perfectly well. Examination of his urine showed it entirely free from albumen.

